# The association between plasma free amino acids and type 2 diabetes mellitus complicated with infection in Chinese patients

**DOI:** 10.1186/s13098-023-01203-w

**Published:** 2024-01-09

**Authors:** Jing-Xi Zhang, Wei-Ming Luo, Bo-Wen Wang, Ru-Tao Li, Qian Zhang, Xiang-Yu Zhang, Zhong-Ze Fang, Zhi-Peng Zhang

**Affiliations:** 1 Department of Pediatric Dentistry, School and Hospital of Stomatology, Tianjin Medical University, No.22, Xinxing Street, Heping District, Tianjin, 300041 China; 2 Department of Toxicology and Sanitary Chemistry, School of Public Health, Tianjin Medical University, No.22, Xinxing Street, Heping District, Tianjin, 300041 China; 3 Tianjin Key Laboratory of Environment, Nutrition and Public Health, Tianjin, 300041 China; 4 General Surgery of Peking University Third Hospital, Beijing, China

**Keywords:** Type 2 Diabetes Mellitus, Chinese, Infection, Plasma free amino acid, Metabolism

## Abstract

**Background:**

Type 2 diabetes mellitus (T2DM), one of the most common public diseases threatening human health, is always accompanied by infection. Though there are still a variety of flaws in the treatment of some infectious diseases, metabolomics provides a fresh perspective to explore the relationship between T2DM and infection. Our research aimed to investigate the association between plasma free amino acids (PFAAs) and T2DM complicated with infection in Chinese patients.

**Methods:**

A cross-sectional study was conducted from May 2015 to August 2016. We retrieved the medical records of 1032 inpatients with T2DM from Liaoning Medical University First Affiliated Hospital and we used mass spectrometry to quantify 23 PFAAs. Infections contained 15 individual categories that could be retrieved from the database. Principal component analysis was used to extract factors of PFAAs. Multi-variable binary logistic regression was used to obtain odds ratios (OR) and their 95% confidence intervals (CI).

**Results:**

Among 1032 inpatients,109 (10.6%) had infectious diseases. Six factors, accounting for 68.6% of the total variance, were extracted. Factor 4 consisted of Glu, Asp and Orn. Factor 5 consisted of Hcy and Pip. After adjusting for potential confounders, factor 4 was positively correlated with T2DM complicated with infection in Chinese T2DM patients (OR: 1.27, 95%CI: 1.06–1.52). Individual Hcy in factor 5 was positively associated with T2DM complicated with infection (OR: 1.33, 95%CI: 1.08–1.64). Furthermore, factor 4 (OR: 1.44, 95%CI: 1.11–1.87), Orn (OR: 1.01, 95%CI: 1.00-1.02) and Hcy (OR: 1.56, 95%CI: 1.14–3.14) were positively associated with bacterial infection in Chinese T2DM patients, while factor 5 (OR: 0.71, 95%CI: 0.50-1.00) was negatively associated with bacterial infection.

**Conclusions:**

Urea cycle-related metabolites (Orn, Asp, Glu) and Hcy were positively associated with T2DM complicated with infection in China. Orn and Hcy were positively associated with bacterial infection in T2DM patients in China.

**Supplementary Information:**

The online version contains supplementary material available at 10.1186/s13098-023-01203-w.

## Introduction

Type 2 diabetes mellitus (T2DM) is one of the most common and complex public diseases. An estimated 529 million people worldwide had T2DM in 2021 [1]. The number of Chinese with diabetes was 145 million and the age-adjusted prevalence was estimated at 10.6% in 2021 [2]. The increased prevalence of T2DM will increase the incidence of infectious diseases and related comorbidities [3]. A landmark prospective study from primary health-care settings showed that, patients with T2DM had a higher risk of lower respiratory tract infections (RTI), urinary tract infections (UTI), bacterial infection and fungal infection of skin and mucosa [4]. A 2018 retrospective cohort study showed patients with diabetes had a higher incidence of all infections compared with controls without diabetes [5]. Patients with T2DM tend to be more susceptible to pathogens than the general population, which is associated with a number of pathways, including prolonged hyperglycemic state resulting in impaired immune response [3], dysfunctional lipid metabolism and neuropathy. Infection can further lead to more serious complications [6] and even increase mortality [7] in patients with T2DM. Diabetes is associated with increased susceptibility, severity and mortality resulting from many infections (e.g. COVID-19, periodontal disease, community-acquired pneumonia, UTI, genital infections, etc.) [8–12]. The most common T2DM co-infection is bacterial infection, which may develop into complicated infection or even need hospitalization [13]. A large number of studies on immunology are currently exploring the mechanisms of T2DM-associated infections, but the treatment of T2DM with certain infections still has many shortcomings. For instance, specific drugs or treatment modalities have not been identified [14], the prognosis of patients with severe and critical illnesses is poor [15] and resistance and side effects from antibiotics have not been addressed [16]. Consequently, it is necessary to explore the influencing factors of infection in T2DM from another perspective so as to predict infection and establish possible biological link between T2DM and infection.

Endogenous small molecule compounds measured by metabolomics can reflect cellular status to some extent [17], providing us with a new perspective to explore the role of a range of metabolites in diseases, such as in infections [6]. Plasma free amino acids (PFAAs) are involved in multiple metabolic process, such as protein synthesis, energy metabolism and serve as signaling molecules. PFAA metabolism has long participated in the regulation of inflammation and pathogen defense in mammals [18], suggesting a close and complex relationship between PFAA metabolism and infection. The metabolism of PFAAs was apparently altered in diabetic patients [19], and certain PFAAs have been reported to reduce the risk of infection in T2DM [20]. Although studies have elucidated potential mechanisms between PFAA metabolism and immune regulation [18], the relationship between PFAA and infection in T2DM was largely unknown. Only one animal study [21] and one cytological study [22] have revealed underlying mechanisms linking some PFAAs to infection in T2DM.

We conducted a hospital-based cross-sectional study of Chinese inpatients with T2DM to explore the global pattern of PFAAs and their role as an indicator of infection risk.

## Methods

### Study population

Previous studies have described the study patients and methods [23]. We retrieved the electronic medical records of 2554 inpatients with available metabolite data from the main electronic database of Liaoning Medical University First Affiliated Hospital (LMUFAH) in Jinzhou, China, from May 2015 to August 2016. All of them paid for the physical tests.

The inclusion criteria were as follows: (1) diagnosed as T2DM; (2) age ≥ 18 years; and (3) 23 PFAAs were available: alanine (Ala), asparagine (Asn), leucine (Leu), phenylalanine (Phe), tryptophan (Trp), tyrosine (Tyr), valine (Val), arginine (Arg), glycine (Gly), proline (Pro), threonine (Thr), citrulline (Cit), glutamine (Gln), histidine (His), lysine (Lys), methionine (Met), serine (Ser), ornithine (Orn), glutamate (Glu), aspartate (Asp), piperamide (Pip), cysteine (Cys) and homocysteine (Hcy);. Exclusion criteria: (1) pregnancy; (2) diabetes secondary to other diseases; (3) incomplete data on height, weight and blood pressure; and (4) mental illness may prevent completing health check. A total of 1032 patients with T2DM who met the inclusion criteria and did not have the exclusion criteria were included in the analysis.

The ethics of the study was approved by the Ethics Committee for Clinical Research of LMUFAH. And due to the retrospective nature of the study, informed consent was waivered, which is consistent with the Declaration of Helsinki.

### Data collection and clinical definition

Demographic and clinical data were retrieved from the main electronic database of the hospital, including age, gender, height, weight, whether smoking or not, whether drinking or not, duration of diabetes, systolic blood pressure (SBP), diastolic blood pressure (DBP), Body Mass Index (BMI), glycated hemoglobin (HbA1c), high-density lipoprotein cholesterol (HDL-C), low-density lipoprotein cholesterol (LDL-C), triglyceride(TG)and drugs use. Detailed use of drugs was recorded, including insulin, oral antidiabetic drugs (acarbose, metformin, sulfonylureas, thiazolidinediones, glinides, and dipeptidyl peptidase-4 inhibitors, DPP-4 inhibitors), angiotensin-converting enzyme inhibitors (ACEIs) or angiotensin receptor blockers (ARBs), other anti-hypertensive drugs (calcium channel blockers, diuretics and beta-blockers), statins, other lipid-lowering drugs and aspirin.

In this study, the diagnosis and classification of T2DM were based on the criteria published by the World Health Organization (WHO) or the population treated with antidiabetic drugs [24]. Specifically, T2DM results from defect(s) in insulin secretion, almost always with a major contribution from insulin resistance. Diabetes Mellitus was defined as the diagnostic fasting plasma (blood) glucose value ≥ 7.0 mmol/l (6.1 mmol/l) or 2-h post glucose load ≥ 11.1 mmol/l (10.0 mmol/l) or both. Infections during 2015–2016 contained 15 different classifications: UTI, lung infection, RTI, endocarditis, oral infection, foot soft tissue infection, bloodstream infection, sepsis, staphylococcus aureus infection, viral infection, fungal infection, systemic infection, infectious shock, infectious fever, diabetic co-infection. Having one or more of these infections was considered a co-infection of T2DM. UTI was defined as asymptomatic bacteriuria, acute uncomplicated cystitis, recurrent cystitis, catheter associated asymptomatic bacteriuria, catheter associated UTI, prostatitis, and pyelonephritis [25]. Lung infection was defined as the presence of an abnormal opacity on chest X-ray and symptoms of respiratory infection such as cough, mucus production, and fever [26]. RTI was defined as any infectious disease of the upper or lower respiratory tract. Upper RTI include the common cold, laryngitis, pharyngitis/tonsillitis, acute rhinitis, acute rhinosinusitis and acute otitis media. Lower RTI include acute bronchitis, bronchiolitis, pneumonia and tracheitis [27]. Bloodstream infection was defined by the growth of a pathogenic organism in culture or by the growth of an atypical organism in combination with symptoms of infection [28]. Sepsis was defined as life-threatening organ failure caused by a dysregulated host response to infection [28].

BMI was calculated by dividing body weight in kilograms by squared height in meters and expressed as kg/m2. The World Health Organization classified BMI for Asians as: Normal weight was defined as 18-24 kg/m2, overweight was defined as BMI ≥ 24.0 kg/m2 but < 28.0 kg/m2, and obesity was defined as BMI ≥ 28.0 kg/m2 [29]. The biochemical parameters measured by the collection of fasting blood at night (at least 8 h fasting) were defined by the American Diabetes Association as follows: HbA1c ≥ 7% (53mmol/mol) was defined as hyperglycaemia, BP ≥ 130/80 mmHg was defined as hypertension, TG ≥ 1.7 mmol/L, LDL-C ≥ 2.6 mmol/L or HDL-C ≥ 1 mmol/L in men and HDL-C ≥ 1.3 mmol/L in women were defined as abnormal lipids [30]. Blood pressure was measured using standard mercury sphygmomanometers and suitably sized adult cuffs on the right arm, after resting in a sitting position for 10 min. Cardiovascular disease (CVD) was defined as having history of coronary heart disease or stroke. Coronary heart disease was defined as having history of angina with abnormal electrocardiogram or on stress test, myocardial infarction, angina coronary artery bypass graft surgery or angioplasty; stroke was defined as nonfatal subarachnoid hemorrhage, intracerebral hemorrhage or other unspecified intracranial hemorrhage and ischemic stroke. Diabetic nephropathy (DN) had following features: persistent albuminuria (or albuminuria excretion rate of > 300 mg/d or 200 mg/min) recorded at least twice within a 3- to 6-month interval, progressive reduction in glomerular filtration rate (GFR) and hypertension. Diabetic retinopathy (DR) was defined as present if any of the following lesions was detected: microaneurysms, retinal hemorrhages, soft exudates, hard exudates, or vitreous hemorrhage. Diabetic peripheral neuropathy (DPN) was defined as the appearance of symptoms and/or signs related to peripheral nerve dysfunction in patients with diabetes, after other causes had been ruled out.

### Laboratory assessments

Metabolomics assessment methods have been published previously [31]. Briefly, all blood samples were collected by finger puncture after 8 h fasting and preserved as dry blood spots. The metabolomic profile of the dry blood spots was measured using mass spectrometry (MS) techniques. MS metabolomic analysis was conducted using an ABSciex4000QTrap system (ABSciex, Framingham, MA, USA). An aqueous 80% acetonitrile solution was used as mobile phase to carry the assayed components. Analyst v1.6.0 software (AB Sciex) was used for system control and data acquisition. ChemoView 2.0.2 (AB Sciex) was used for data preprocessing. Isotope-labeled internal standard samples of 23 PFAAs (NSK-A) were purchased from Cambridge Isotope Laboratories (Tewksbury, MA, USA), while standard samples of PFAAs were purchased from Chrom Systems (Grafelfing, Germany).

### Statistical analysis

IBM SPSS Statistics 26 was used for statistical analysis. All p-values were two-tailed. P < 0.05 was considered as statistically significant. When the analysis variables were continuous, normality tests were performed using P-P plots or Q-Q plots. When comparing variables in the two groups, continuous variables that conformed to a normal distribution were expressed as mean ± standard deviation (SD), tested for chi-square and compared using the Student’s t-test. Otherwise, they were expressed as median with interquartile range (IQR) and compared using the Wilcoxon signed rank test. Comparisons of categorical data between the two groups were carried out by Chi-square test or Fisher’s exact test and expressed as number (percentile). The false discovery rate (FDR) was calculated for multiple comparisons of 23 PFAAs and q < 0.05 was defined as statistically significant.

In order to describe the association of many amino acids with a few factors, factor analysis can group several closely related amino acids into the same category, reducing a large number of amino acids to a smaller number of factors. Consequently, factor analysis was used to deal with multiple comparisons and extract common factors from the 23 PFAAs. The Kaiser-Meyer-Olkin (KMO) and Bartlett sphericity tests were used to assess the applicability of factor analysis [32]. A KMO coefficient around 0.8 was considered as excellent. Principal component analysis (PCA) was used to reduce the dimension of 23 PFAAs and extract common factors. Orthogonal rotation (varimax) was used to better interpret the results. Individual PFAA that load most heavily for a factor were used as the relevant components of the factor. The number of PFAA factors was determined by eigenvalue, communality and the scree plot: eigenvalue > 1, communalities ≥ 50% and number of factors located on the steep slope of scree plot, which is a line graph of the eigenvalues of the factors in factor analysis.

Multivariate binary logistic regression was used to predict the odds ratios (ORs) and their 95% confidence intervals (CIs) of the extracted factors and individual PFAAs for T2DM complicated with infection and bacterial infection. Patients’ demographics data, lifestyle, past medical history, and clinical variables were collected as confounding factors and included in the regression model. In addition, we also included clinically common diabetic complications such as CVD, DN, DR and DPN in the regression model to exclude the effect of other complications on results. A structured adjustment scheme was used to control for confounding effects of demographic and clinical variables. First of all, we used uni-variable analysis to obtain an unadjusted OR (model 1). Secondly, we used diverse models to adjust for different confounders to obtain the corresponding ORs. Model 2 was adjusted for age, gender, smoking, diabetes duration, weight, height; model 3 was further adjusted for SBP, DBP, HbA1c, HDL-C, LDL-C, and TG; model 4 included aspirin, antidiabetic drugs, lipid lowering drugs, antihypertensive drugs, common complications such as CVD, DN, DR and DPN in addition to the previously adjusted variables.

In order to explore the correlations between PFAAs and inflammatory indicators, we used Pearson’s correlation coefficient if the continuous variables conformed to a normal distribution. Otherwise, we used Spearman’s correlation coefficient. P value < 0.05 was considered as statistically significant.

## Results

### Clinical characteristics of study subjects

A total of 1032 subjects were included in this study and were divided into two groups: the group with infection in T2DM (n = 109), and the group without infection in T2DM (n = 923). The mean age of the study subjects was 57.2 (SD: 13.82) years, and the median duration of diabetes was 5 (IQR: 0–10) years.

In the group with infection in T2DM, 27 patients had UTI, 38 patients had lung infection, 11 patients had RTI, 1 patient had endocarditis, 3 patients had oral infection, 9 patients had foot soft tissue infection, 2 patients had bloodstream infection, 4 patients had sepsis, 2 patients had staphylococcus aureus infection, 10 patients had viral infection, 2 patients had fungal infection, 1 patient had systemic infection, 3 patients had infectious shock, 16 patients had infectious fever, 2 patients had diabetic co-infection. Organ systems where bacterial infections predominate included UTI, lung infection, RTI, endocarditis, oral infection, foot soft tissue infection, bloodstream infection, sepsis and staphylococcus aureus infection. 98 patients with diabetes had bacterial infection.

As shown in Table 1, two groups were statistically significant for age, sex, BMI, smoking, weight, height, CVD and DR. Specifically, the T2DM group with infection was older, and had more women and fewer smokers. BMI, weight, height in the T2DM group with infection were lower than the control group. Besides, The concentrations of Asn and Pro in the T2DM patients with infection were lower than those in the control group, but the concentrations of Hcy and Phe were higher than those in the control group. (Table 2). Difference in other PFAAs between the two groups was not statistically significant. The prevalence of CVD was higher in the T2DM co-infection group than in the control group, but the prevalence of DR was lower in the T2DM co-infection group than in the control group.

**Table 1:** Clinical characteristics of patients with T2DM according to infectious disease status

Variables	The group with infection in T2DM (n = 109) Mean/number (SD or %)	The group without infection in T2DM (n = 923) Mean/number (SD or %)	P
N	109(10.6)	923(89.4)	
Age, years	62.61 ± 14.02	56.60 ± 13.66	< 0.0001*
Duration of diabetes, years	7.00(1.00, 10.50)	5.00(0.00, 10.00)	0.140***
Male Gender	45(41.3)	504(54.6)	0.008**
BMI, kg/m^2^	19.16 ± 1.46	26.02 ± 3.37	< 0.0001*
BMI categories			< 0.0001**
< 24	109(100)	272(29.5)	
24 ≥ and < 28	0(0)	430(46.6)	
≥ 28	0(0)	221(23.9)	
Height, cm	164.97 ± 8.11	166.65 ± 8.18	0.042*
Weight, kg	67.82 ± 13.51	70.64 ± 13.10	0.034*
SBP, mmHg	140.64 ± 28.18	140.38 ± 23.46	0.916*
DBP, mmHg	82.00 ± 15.69	82.51 ± 13.25	0.710*
Smoking	24(22.0)	307(33.3)	0.017**
Drinking	22(20.2)	268(29.0)	0.052**
HbA1c, %(mmol/mol)	9.75 ± 2.07(83 ± 22)	9.58 ± 1.83(81 ± 20)	0.389*
HbA1c categories			0.243**
< 7%(53mmol/mol)	6(5.5)	71(7.7)	
7%~8%(53 ~ 64mmol/mol)	17(94.5)	89(92.3)	
≥ 8%(64mmol/mol)	46(42.2)	402(43.6)	
TG, mmol/L	1.98 ± 1.20	2.06 ± 1.35	0.548*
TG categories			0.593**
TG < 1.7	43(39.4)	340(36.8)	
TG ≥ 1.7	66(60.6)	583(63.2)	
LDL-C, mmol/L	2.82 ± 0.96	2.90 ± 0.84	0.374*
LDL-C categories			0.217**
LDL-C < 2.6	38(34.9)	269(29.1)	
LDL-C ≥ 2.6	71(65.1)	654(70.9)	
HDL-C, mmol/L	1.04 ± 0.26	1.08 ± 0.30	0.124*
HDL-C categories			0.987**
< 1 in male or < 1.3 in female	82(75.2)	695(75.3)	
≥ 1 in male or ≥ 1.3 in female	27(24.8)	228(24.7)	
Antidiabetic drugs	88(80.7)	779(84.4)	0.323**
OAD	52(47.8)	517(56.0)	0.099**
DPP-4 inhibitors	0(0)	6(0.7)	1.000**
Insulin	77(70.6)	695(75.3)	0.290**
Aspirin	35(32.1)	288(31.2)	0.847**
Lipid lowering drugs	44(40.4)	344(37.3)	0.528**
Statin	44(40.4)	326(35.3)	0.299**
fibrates	1(0.9)	20(2.2)	0.504**
OLLD	0(0)	5(0.5)	0.661**
Antihypertensive drugs	48(44.0)	365(39.5)	0.365**
Angiotensin drugs	30(27.5)	228(24.7)	0.520**
OAHD	40(36.7)	269(29.1)	0.103**
CVD	60(55.0)	294(31.9)	< 0.0001**
DR	10(9.2)	152(16.5)	0.048**
DN	19(17.4)	169(18.3)	0.822**
DPN	14(12.8)	83(9.0)	0.193**

Data are represented as means ± standard deviation, n(%), median (interquartile range)

*BMI* Body Mass Index, *SBP* systolic blood pressure, *DBP* diastolic blood pressure, *HbA1c* glycated haemoglobin, *HDL-C* high-density lipoprotein cholesterol, *LDL-C* low-density lipoprotein cholesterol, *TG* triglyceride, *OAD* oral antidiabetic drugs, *DPP-4* dipeptidyl peptidase-4, *OLLD* other lipid lowering drugs, *OAHD* other antihypertensive drugs, *CVD* cardiovascular disease, *DR* diabetic retinopathy, *DN* diabetic nephropathy, *DPN* diabetic peripheral neuropathy

*P-values for comparisons between groups derived by Student’s t-test

**P-values for comparisons between groups derived by Chi squared test

***P-values for comparisons between groups derived by Wilcoxon Signed Rank Test

**Table 2:** PFAA profile and identified factors by infectious status

Variables	The group with infection in T2DM (n = 109)	The group without infection in T2DM (n = 923)	p	q
	Mean ± SD/Median(IQR)	Mean ± SD/Median (IQR)		
Phe, µmol/L	55.0 ± 28.0	47.1 ± 14.4	0.000	**0.000**
Hcy, µmol/L	8.0 ± 0.9	7.6 ± 1.1	0.001	**0.007**
Pro, µmol/L	416.0 ± 200.5	480.1 ± 201.5	0.002	**0.012**
Asn, µmol/L	71.3 ± 20.4	78.6 ± 24.4	0.003	**0.014**
Cit, µmol/L	19.7 ± 7.8	21.8 ± 10.4	0.039	0.120
Val, µmol/L	133.0 ± 42.0	142.0 ± 41.0	0.039	0.120
Leu, µmol/L	124.5 ± 39.8	134.0 ± 47.5	0.044	0.120
Gly, µmol/L	234.3 ± 99.1	216.1 ± 89.6	0.047	0.120
His, µmol/L	71.7 ± 46.5	63.4 ± 44.6	0.068	0.142
Asp, µmol/L	32.6 ± 15.5	30.2 ± 12.7	0.077	0.148
Orn, µmol/L	18.0(12.4–24.3)	17.4(13.2–23.5)	0.613	0.742
Ala, µmol/L	122.7 ± 54.9	130.0 ± 43.0	0.108	0.191
Met, µmol/L	17.4 ± 6.5	18.4 ± 6.6	0.12	0.197
Glu, µmol/L	109.7 ± 36.4	104.1 ± 36.3	0.13	0.199
Gln, µmol/L	6.1(4.7–8.5)	7.0(5.2–9.3)	0.057	0.131
Pip, µmol/L	126.0(97.5-177.3)	128.0(94.5-174.2)	0.919	0.919
Trp, µmol/L	49.7 ± 14.3	48.4 ± 14.1	0.358	0.511
Thr, µmol/L	27.2 ± 14.7	26.3 ± 9.8	0.383	0.511
Arg, µmol/L	11.9 ± 8.0	12.7 ± 9.1	0.400	0.511
Tyr, µmol/L	47.6 ± 17.9	48.4 ± 16.3	0.669	0.769
Lys, µmol/L	143.3 ± 68.6	141.1 ± 78.5	0.779	0.814
Ser, µmol/L	51.4(41.9–66.2)	51.7(43.1–64.2)	0.761	0.814
Cys, µmol/L	1.5(1.2-2.0)	1.2(0.9–1.7)	0.000	**0.000**

*SD* standard deviation, *IQR* interquartile range, *Ala* Alanine, *Asn* Asparagine, *Leu* Leucine, *Phe* Phenylalanine, *Trp* Tryptophan, *Tyr* Tyrosine, *Val* Valine, *Arg* Arginine, *Gly* Glycine, *Pro* Proline, *Thr* Threonine, *Cit* Citrulline, *Gln* Glutamine, *His* Histidine, *Lys* Lysine, *Met* Methionine, *Ser* Serine, *Orn* Ornithine, *Glu* Glutamate, *Asp* aspartate, *Pip* Piperamide, *Cys* Cysteine, *Hcy* Homocysteine

### Extracted factors of PFAAs

**Fig. 1:**
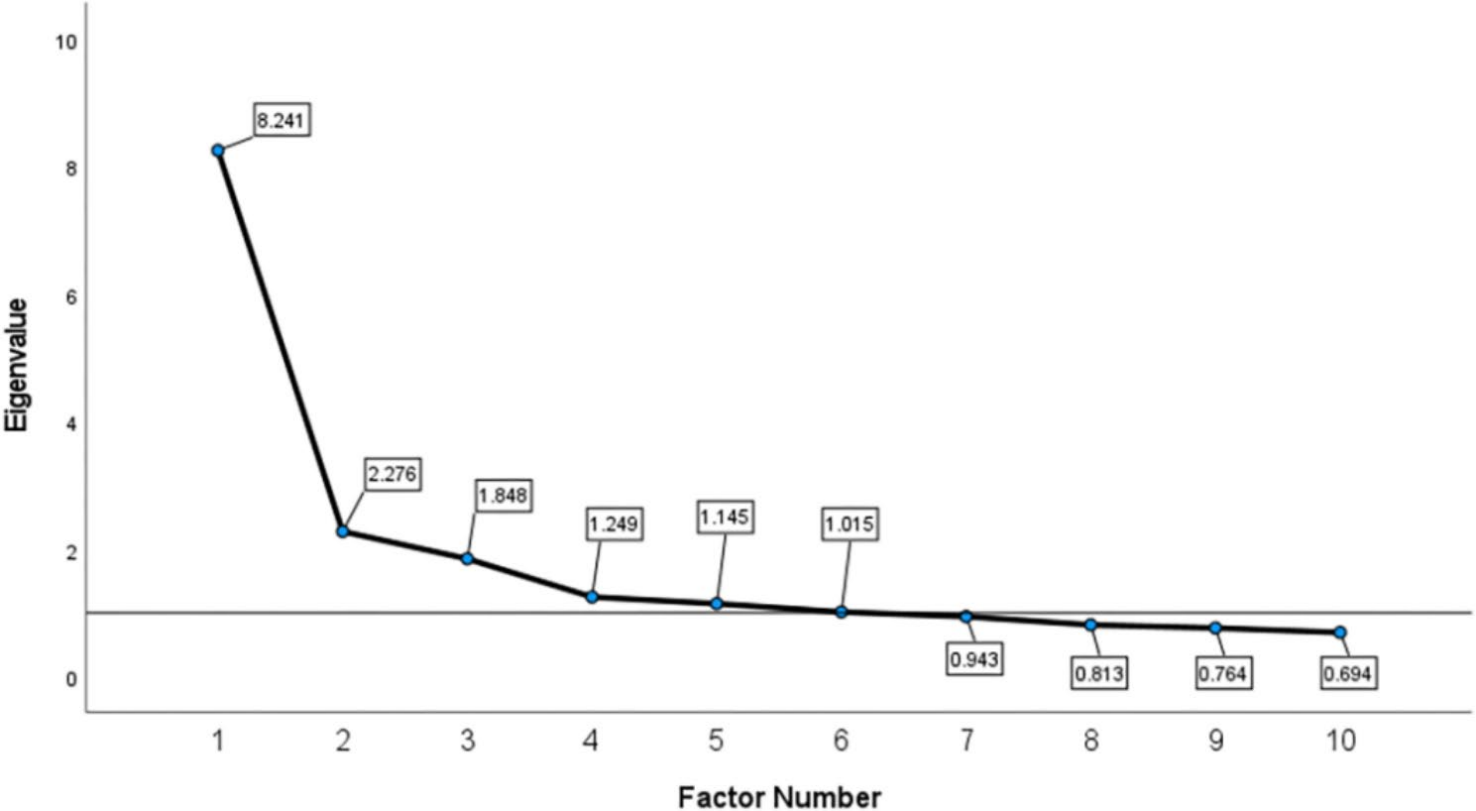
Scree plot: the horizontal axis is the number of factors, and the vertical axis is the eigenvalue of factors

The KMO coefficient was 0.860 and P value of Bartlett sphericity test was < 0.0001, so the results of the factor analysis were acceptable. Factors 1–6 had eigenvalues greater than 1 and were situated on the steep slope of scree plot (Fig. 1). Therefore, we extracted 6 factors that explained 68.6% of the total variance. The loadings of PFAAs after rotation are shown in Table 3. Factor 1 included Asn, Leu, valine, Tyr, Met, Phe, Trp, Ala and Thr; factor 2 included Gly, Arg, Thr, Pro, Ser and His; factor 3 included Gln, Lys and His; factor 4 included Orn, Asp and Glu; factor 5 included Hcy and Pip; factor 6 included Cys.

**Table 3:** Factor and their loadings derived by 23 PFAA metabolites

Variables	Factor1	Factor2	Factor3	Factor4	Factor5	Factor6
Asn	**0.914**	0.080	0.010	0.042	0.087	0.024
Leu	**0.911**	0.161	0.030	0.058	0.035	-0.011
Val	**0.889**	0.124	0.034	0.033	0.010	0.022
Tyr	**0.755**	0.262	0.057	0.214	0.047	0.053
Met	**0.649**	0.170	0.448	0.130	-0.056	0.003
Phe	**0.590**	0.475	0.000	0.156	0.056	0.054
Trp	**0.525**	0.465	0.148	0.410	0.096	-0.060
Ala	**0.518**	0.421	0.206	0.057	0.143	-0.074
Gly	0.171	**0.811**	0.016	0.205	0.112	0.052
Arg	0.236	**0.654**	0.118	-0.042	-0.088	-0.086
Thr	**0.511**	**0.575**	0.138	0.277	-0.032	0.205
Pro	0.417	**0.530**	0.266	-0.342	0.160	-0.028
Ser	0.345	**0.519**	-0.083	0.431	-0.026	0.121
Cit	0.054	0.469	0.194	0.274	-0.017	-0.050
Gln	0.078	0.023	**0.905**	0.089	0.203	0.094
Lys	0.077	0.149	**0.897**	0.053	-0.093	0.003
His	0.105	**0.529**	**0.584**	0.148	-0.056	0.041
Orn	0.002	-0.035	0.121	**0.821**	-0.102	0.011
Asp	0.226	0.310	0.029	**0.686**	0.039	0.015
Glu	0.236	0.391	0.155	**0.615**	0.293	-0.041
Hcy	-0.045	0.078	0.120	0.177	**-0.830**	-0.115
Pip	0.081	0.106	0.195	0.225	**0.521**	-0.131
Cys	0.049	-0.001	0.091	0.008	0.002	**0.955**

Six factors were extracted based on eigenvalues > 1, scree plot, and cumulative variance by principal component analysis.Individual PFAA with absolute loading > 0.50 was considered as relevant components of the identified factors and marked in black

*Ala* Alanine, *Asn* Asparagine, *Leu* Leucine, *Phe* Phenylalanine, *Trp* Tryptophan, *Tyr* Tyrosine, *Val* Valine, *Arg* Arginine, *Gly* Glycine, *Pro* Proline, *Thr* Threonine, *Cit* Citrulline, *Gln* Glutamine, *His* Histidine, *Lys* Lysine, *Met* Methionine, *Ser* Serine, *Orn* Ornithine, *Glu* Glutamate, *Asp* aspartate, *Pip* Piperamide, *Cys* Cysteine, *Hcy* Homocysteine

### Association of extracted factors with infectious event in T2DM

Univariate analysis showed (model 1) that factor 1 and factor 4 were associated with infection in T2DM patients. Factor 4 (OR: 1.30, 95%CI: 1.10–1.54) was positively correlated, and factor 1 (OR: 0.78, 95%CI: 0.63–0.97) was negatively correlated. After adjusting for gender, age, diabetes duration, weight, height (Model 2), factors 4 and 5 were associated with infection in T2DM patients. Factor 4 (OR: 1.29, 95%CI: 1.08–1.53) was positively correlated and factor 5 (OR: 0.78, 95% CI: 0.63–0.97) was negatively correlated. After further adjustment for SBP, DBP, HbA1c, HDL-C, LDL-C, and TG (model 3), only factor 4 (OR: 1.27, 95%CI: 1.07–1.51) remained positively associated with infection in T2DM. After final adjustment for drug use and common complications, factor 4 (OR: 1.27, 95%CI: 1.06–1.52) was steadily associated with infection (Table 4).

**Table 4:** Uni-variable and multi-variable association of metabolomic factors with infectious event

Factor	Model	OR	95%CI	q
Factor 1	Model 1	0.78	0.63–0.97	0.041
	Model 2	0.86	0.68–1.08	0.303
	Model 3	0.86	0.68–1.08	0.300
	Model 4	0.88	0.69–1.11	0.432
Factor 2	Model 1	1.16	0.96–1.40	0.408
	Model 2	1.15	0.95–1.40	0.303
	Model 3	1.18	0.97–1.43	0.101
	Model 4	1.13	0.92–1.38	0.258
Factor 3	Model 1	0.88	0.68–1.15	0.522
	Model 2	0.80	0.60–1.07	0.137
	Model 3	0.82	0.62–1.09	0.300
	Model 4	0.86	0.64–1.14	0.432
Factor 4	Model 1	1.30	1.10–1.54	0.006
	Model 2	1.29	1.08–1.53	0.012
	Model 3	1.27	1.07–1.51	0.018
	Model 4	1.27	1.06–1.52	0.033
Factor 5	Model 1	0.83	0.67–1.02	0.080
	Model 2	0.78	0.63–0.97	0.041
	Model 3	0.81	0.65–1.01	0.093
	Model 4	0.82	0.65–1.03	0.135
Factor 6	Model 1	1.04	0.90–1.20	0.638
	Model 2	1.05	0.91–1.20	0.502
	Model 3	1.04	0.91–1.19	0.568
	Model 4	1.06	0.92–1.22	0.441

Model 1: Uni-variable model; Model 2: Multi-variable model, adjusted for age, gender, smoking, diabetes duration, weight, height; Model 3: Multi-variable model, further adjusted for SBP, DBP, HbA1c, HDL-C, LDL-C, and TG; Model 4: Multi-variable model, further adjusted for aspirin, antidiabetic drugs, lipid lowering drugs, antihypertensive drugs, cardiovascular disease, diabetic nephropathy, diabetic retinopathy and diabetic peripheral neuropathy. *OR* odds ratio, *CI* confidence interval

### Association between individual amino acids in factor 4, factor 5 and infectious event in T2DM

Hcy was positively associated with T2DM co-infection (OR: 1.33, 95%CI: 1.08–1.64). But Orn, Asp, Glu or Pip were not significantly associated with T2DM co-infection (Table 5).

**Table 5:** Odds ratio of PFAA in factor 4 and factor 5 for infectious event in T2DM

Factor	PFAA	Model	OR	95%CI	P
Factor 4	Orn	Model 1	1.01	1.00-1.01	0.107
		Model 2	1.01	1.00-1.01	0.127
		Model 3	1.01	1.00-1.01	0.154
		Model 4	1.01	1.00-1.01	0.136
Factor 4	Asp	Model 1	1.01	1.00-1.03	0.078
		Model 2	1.01	1.00-1.03	0.071
		Model 3	1.01	1.00-1.03	0.059
		Model 4	1.01	1.00-1.03	0.119
Factor 4	Glu	Model 1	1.00	1.00-1.01	0.128
		Model 2	1.00	1.00-1.01	0.197
		Model 3	1.00	1.00-1.01	0.176
		Model 4	1.00	1.00-1.01	0.262
Factor 5	Hcy	Model 1	1.37	1.13–1.66	0.001
		Model 2	1.37	1.13–1.66	0.002
		Model 3	1.33	1.09–1.62	0.004
		Model 4	1.33	1.08–1.64	0.009
Factor 5	Pip	Model 1	1.00	0.99-1.00	0.222
		Model 2	1.00	0.99-1.00	0.643
		Model 3	1.00	0.99-1.00	0.463
		Model 4	1.00	0.99-1.00	0.624

Model 1: Uni-variable model; Model 2: Multi-variable model, adjusted for age, gender, smoking, diabetes duration, weight, height; Model 3: Multi-variable model, further adjusted for SBP, DBP, HbA1c, HDL-C, LDL-C, and TG; Model 4: Multi-variable model, further adjusted for aspirin, antidiabetic drugs, lipid lowering drugs, antihypertensive drugs, cardiovascular disease, diabetic nephropathy, diabetic retinopathy and diabetic peripheral neuropathy. *OR* odds ratio, *CI* confidence interval, *PFAA* plasma free amino acid, *Orn* Ornithine, *Glu* Glutamate, *Asp* Aspartate, *Pip* Piperamide, *Hcy* Homocysteine

### Association between extracted factors and bacterial infection in T2DM

Univariate analysis showed (model 1) that factor 4, factor 5 were associated with bacterial infection in T2DM patients. Factor 4 (OR: 1.48, 95%CI: 1.18–1.86) was positively correlated, and factor 5 (OR: 0.70, 95%CI: 0.51–0.96) was negatively correlated. After adjusting for gender, age, smoking, diabetes duration, weight, height (Model 2), factors 4, factor 5 were still associated with bacterial infection in T2DM patients. Factor 4 (OR: 1.47, 95%CI: 1.16–1.87) was positively correlated, and factor 5 (OR: 0.65, 95%CI: 0.47–0.89) was negatively correlated. After further adjustment for SBP, DBP, HbA1c, HDL-C, LDL-C, and TG (model 3), the results remained unchanged. Factor 4 (OR: 1.49, 95%CI: 1.17–1.90) was positively correlated, and factor 5 (OR: 0.68, 95%CI: 0.49–0.93) was negatively correlated. After final adjustment for drug use and common complications, Factor 4 (OR: 1.44, 95%CI: 1.11–1.87) was positively correlated, and factor 5 (OR: 0.71, 95%CI: 0.50-1.00) was negatively correlated. (Table 6).

**Table 6:** Uni-variable and multi-variable association of metabolomic factors with bacterial infection

Factor	Model	OR	95%CI	q
Factor 1	Model 1	0.74	0.54–1.03	0.213
	Model 2	0.80	0.57–1.12	0.278
	Model 3	0.81	0.57–1.13	0.321
	Model 4	0.84	0.58–1.20	0.333
Factor 2	Model 1	0.93	0.69–1.27	0.662
	Model 2	0.92	0.67–1.27	0.616
	Model 3	0.91	0.65–1.28	0.596
	Model 4	0.85	0.60–1.20	0.354
Factor 3	Model 1	0.79	0.53–1.19	0.393
	Model 2	0.68	0.44–1.06	0.278
	Model 3	0.71	0.46–1.11	0.321
	Model 4	0.72	0.45–1.16	0.354
Factor 4	Model 1	1.48	1.18–1.86	0.002
	Model 2	1.47	1.16–1.87	0.003
	Model 3	1.49	1.17–1.90	0.003
	Model 4	1.44	1.11–1.87	0.006
Factor 5	Model 1	0.70	0.51–0.96	0.038
	Model 2	0.65	0.47–0.89	0.007
	Model 3	0.68	0.49–0.93	0.027
	Model 4	0.71	0.50-1.00	0.047
Factor 6	Model 1	1.66	0.65–4.19	0.287
	Model 2	2.00	0.80-5.00	0.136
	Model 3	1.98	0.77–5.10	0.155
	Model 4	2.11	0.79–5.68	0.354

Model 1: Uni-variable model; Model 2: Multi-variable model, adjusted for age, gender, smoking, diabetes duration, weight, height; Model 3: Multi-variable model, further adjusted for SBP, DBP, HbA1c, HDL-C, LDL-C, and TG; Model 4: Multi-variable model, further adjusted for aspirin, antidiabetic drugs, lipid lowering drugs, antihypertensive drugs, cardiovascular disease, diabetic nephropathy, diabetic retinopathy and diabetic peripheral neuropathy. *OR* odds ratio, *CI* confidence interval

### Association between individual amino acids in factor 4, factor 5 and bacterial infection in T2DM

Orn and Hcy were positively correlated with bacterial infection in T2DM patients in four models. Their ORs and 95% CIs were detailed in Table 7.

**Table 7:** Odds ratio of PFAA in factor 4 and factor 5 for bacterial infectious event in T2DM

Factor	PFAA	Model	OR	95%CI	P
Factor 4	Orn	Model 1	1.01	1.00-1.02	0.009
		Model 2	1.01	1.00-1.02	0.012
		Model 3	1.01	1.00-1.03	0.008
		Model 4	1.01	1.00-1.02	0.034
Factor 4	Asp	Model 1	1.02	1.00-1.04	0.158
		Model 2	1.02	1.00-1.04	0.126
		Model 3	1.01	1.00-1.04	0.104
		Model 4	1.01	0.99–1.04	0.237
Factor 4	Glu	Model 1	1.00	0.99–1.01	0.963
		Model 2	1.00	0.99–1.01	0.926
		Model 3	1.00	0.99–1.01	0.921
		Model 4	1.00	0.99–1.01	0.888
Factor 5	Hcy	Model 1	1.55	1.18–2.03	0.002
		Model 2	1.62	1.23–2.14	< 0.001
		Model 3	1.57	1.18–2.08	0.002
		Model 4	1.56	1.14–2.14	0.005
Factor 5	Pip	Model 1	1.00	0.99-1.00	0.244
		Model 2	1.00	0.99-1.00	0.491
		Model 3	1.00	0.99-1.00	0.362
		Model 4	1.00	0.99-1.00	0.507

Model 1: Uni-variable model; Model 2: Multi-variable model, adjusted for age, gender, smoking, diabetes duration, weight, height; Model 3: Multi-variable model, further adjusted for SBP, DBP, HbA1c, HDL-C, LDL-C, and TG; Model 4: Multi-variable model, further adjusted for aspirin, antidiabetic drugs, lipid lowering drugs, antihypertensive drugs, cardiovascular disease, diabetic nephropathy, diabetic retinopathy and diabetic peripheral neuropathy. *OR* odds ratio, *CI* confidence interval, *PFAA* plasma free amino acid, *Orn* Ornithine, *Glu* Glutamate, *Asp* Aspartate, *Pip* Piperamide, *Hcy* Homocysteine

### Sensitivity analysis

After median imputation and multiple imputation of missing values in HDL-C, LDL-C and TG (n = 291), the effect sizes of factor 4, factor 5 (Table S1) and individual PFAA (Table S2) for T2DM complicated with infection remained stable and significant in uni-variable and multi-variable analyses.

### Correlation between individual amino acids in factor 4, factor 5 and inflammatory indicators in T2DM with infection

The results of Pearson’s and Spearman’s correlation analysis were shown in the supplementary material (Table S3).

## Discussion

Metabolomics is becoming increasingly valuable as a new biomarker for predicting disease [33]. To elucidate the possible mechanisms underlying the emergence of T2DM complicated with infection in Chinese patients, we explored the overall pattern of different PFAAs and their role as an indicator of T2DM complicated with infection in the Chinese cohort. The one key finding of our study was that factor 4 was positively associated with T2DM complicated with infection and independent of traditional risk factors. Surprisingly, the individual Orn, Asp and Glu in factor 4 were not significantly associated with T2DM complicated with infection. Furthermore, factor 4 and individual Orn were associated with the bacterial infection, respectively. Considering that factor 4 was associated with infection in T2DM, but not individual PFAA, we speculated that the metabolic pathways in which Orn, Asp and Glu involved were critical for immune regulation.

Common non-essential PFAAs Orn, Asp, and Glu all take part in the Orn cycle (urea cycle). Urea cycle is a pivotal PFAA metabolic pathway whose dysregulation has been observed in a large number of diseases, such as infections [34], cancer [35] and metabolic diseases [36]. It is widely known that ammonia is normally converted to urea through urea cycle. The dysregulation of urea cycle further leads to the accumulation of ammonia. On the one hand, accumulating ammonia and some metabolites, for example, Orn, are conductive to redox dysfunction [37]. Oxidative stress is one of the causes of inflammation [38]. On the other hand, Orn, Asp and Glu enter the tricarboxylic acid (TCA) cycle in different forms [39–41]. When the content of Orn, Asp, and Glu increases, it may indicate that the activity of the urea cycle is reduced and accumulated ammonia inhibited TCA cycle activity [42]. A detailed review has revealed that TCA cycle intermediate metabolites play a key role in the pro-inflammatory/anti-inflammatory homeostasis [43]. So TCA cycle controls function and plasticity of immune responses [44]. Various studies have found that the interruption of TCA cycle also supports a shift to a pro-inflammatory phenotype [45]. We found that factor 4, composed of urea cycle-related metabolites (Orn, Asp, Glu), was positively associated with T2DM complicated with infection in the current Chinese population. Their risk-associated mechanism may be inflammatory response, oxidative stress and increased pathogen susceptibility due to dysregulation of urea cycle and decreased TCA cycle activity. In terms of bacterial infections, a study published in NATURE showed that Orn can increase susceptibility to bacteria and enhance pathogenesis, which supported our results [46]. However, many authors reckoned that Orn could strengthen the host’s defenses against infection [41], which was contrary to our results. Two factors could account for the discrepancy between our research and previous studies [41]:1) The association of Orn and infection in the general population is distinct from that in the diabetic population; 2) The heterogeneity of infection subtypes, which means the type of pathogen, may be responsible for the difference. Consequently, future research should focus further on the regulation of related PFAAs metabolism, which may contribute to preventing the infection in T2DM.

And the other point was that we found Hcy in factor 5 was also positively correlated with infection, especially the bacterial infection. Hcy, a sulfur-containing PFAA derived from Met, is one of the nonessential PFAAs. Elevated plasma Hcy concentration is now recognized as an independent risk factor for cardiovascular disease, while a limited number of studies have shown that Hcy is associated with infectious event [47]. Dierkes et al. [48] have suggested that Hcy is not only strongly correlated with insulin resistance, but also stimulates pro-inflammatory cytokine secretion. Previous studies drew the similar conclusions. A retrospective cohort study based on healthy Chinese population have shown that bacterial infection can elevate serum Hcy concentration [49]. An Egyptian study held the view that Hcy could be a new diagnostic marker for spontaneous bacterial peritonitis [50]. However, contrary to the association between Hcy and T2DM complicated with infection, factor 5 was negatively associated with T2DM complicated with infection in terms of individual PFAA. This might be due to the interference of another amino acid in factor 5, Pip, which has been proved to be an adjunct to antibiotics [51]. We suggested that the association between Hcy and infection in T2DM may be due to the destruction of vascular endothelial cells, increased individual susceptibility to pathogenic microorganisms and increased inflammatory responses. Since previous studies have shown that the sulfation factor-like effects of Hcy are directly toxic to endothelial cells [52] and damage endothelial cell-dependent vasodilation [48]. Destruction of endothelial cells results in impaired barrier function in the first place. Secondly, the coagulation function is affected. Once the thrombus is difficult to form, microorganisms can easily spread [53]. Otherwise, hyperhomocysteinemia-induced oxidative stress and inflammation are both important pathogenesis of infection [54]. However, few metabonomic biomarkers are available to detect the severity and progression rate of infection in T2DM. Our study suggested that if these findings could be replicated in cohort studies, Hcy might be a candidate marker for future risk scoring in Chinese T2DM patients with infection.

Our findings have potential public and clinical health implications. It’s widely known that infection is one of the common complications in T2DM patients. Our findings explored the possible mechanism of infection in T2DM and provided clues to its etiology. In addition, whether PFAAs are involved in the urea cycle in T2DM patients is largely unknown, particularly in China [55]. But our research showed the association between urea cycle-related amino acids and infection in T2DM. There were several limitations in our study: first, our study was a cross-sectional survey. We couldn’t establish a causal relationship as a result of the missing duration of infection. Second, we didn’t collect information on diet, physical activity and socioeconomic variables (education and income). In our study, patients willing to pay for the physical tests might have better education and income. Future investigations should include subjects with different characteristics. Third, we did not measure other inflammatory factor levels involved in the immune regulation, which should be included in future studies to better explain the association between metabonomics and infection in T2DM. Fourth, there was a small number of patients with viral or fungal infections, and future studies could pay more attention to these two types of infection.

In conclusion, we found that factor 4 composed of urea cycle-related metabolites (Orn, Asp, Glu), and Hcy were associated with infection in Chinese hospitalized T2DM patients. Orn, Hcy, factor 4 and factor 5 were associated with an bacterial infection in Chinese hospitalized T2DM patients. More high-quality epidemiological and experimental studies are needed to confirm and explain our findings in the future.

### Electronic supplementary material

Below is the link to the electronic supplementary material.

Supplementary Material 1. Association of metabonomic factors with infectious event after median imputation and multiple imputation to missing value of triglyceride, high-density lipoprotein cholesterol and low-density lipoprotein cholesterol

## Data Availability

The datasets generated for this study can be found in Metabolights, with the unique identifier MTBLS1427, accessible via http://www.ebi.ac.uk/metabolights/MTBLS1427.
