# Supplementary material for: The association between plasma free amino acids and type 2 diabetes mellitus complicated with infection in Chinese patients
Source: Diabetol Metab Syndr. 2024 Jan 9;16:9. doi: 10.1186/s13098-023-01203-w (PMC10775586; doi:10.1186/s13098-023-01203-w)
Supplement: Supplementary file 1 — Supplementary Material 1. Association of metabonomic factors with infectious event after median imputation and multiple imputation to missing value of triglyceride, high-density lipoprotein cholesterol and low-density lipoprotein cholesterol [file 13098_2023_1203_MOESM1_ESM.docx]

Supplementary Material

The Association Between Plasma Free Amino Acids and Type 2 Diabetes Mellitus complicated with infection in Chinese Patients

Jing-Xi Zhang^1†^, Wei-Ming Luo^2†^, Bo-Wen Wang^2^, Ru-Tao Li^1^, Qian Zhang^2^, Xiang-Yu Zhang^1*^and Zhong-Ze Fang^2,3*^

*** Correspondence:**

Zhong-Ze Fang: fangzhongze@tmu.edu.cn

Xiang-Yu Zhang: [xzhang04@tmu.edu.cn](mailto:xzhang04@tmu.edu.cn)

Table S1 Association of metabonomic factors with infectious event after median imputation and multiple imputation to missing value of triglyceride, high-density lipoprotein cholesterol and low-density lipoprotein cholesterol

|  |  | | | Median imputation | | | | Multiple imputation | | |
| --- | --- | --- | --- | --- | --- | --- | --- | --- | --- | --- |
| Factor | | Model | OR | | 95% CI | P | OR | | 95% CI | P |
| Factor 1 | | Model 1 | 0.78 | | 0.63-0.97 | 0.027 | 0.78 | | 0.63-0.97 | 0.027 |
|  |  | Model 2 | 0.86 | | 0.68-1.08 | 0.202 | 0.86 | | 0.68-1.08 | 0.202 |
|  |  | Model 3 | 0.86 | | 0.68-1.09 | 0.205 | 0.86 | | 0.68-1.09 | 0.210 |
|  |  | Model 4 | 0.87 | | 0.69-1.10 | 0.237 | 0.87 | | 0.69-1.11 | 0.264 |
| Factor 2 | | Model 1 | 1.16 | | 0.96-1.40 | 0.136 | 1.16 | | 0.96-1.40 | 0.136 |
|  |  | Model 2 | 1.15 | | 0.95-1.40 | 0.160 | 1.15 | | 0.95-1.40 | 0.160 |
|  |  | Model 3 | 1.18 | | 0.97-1.43 | 0.102 | 1.20 | | 0.98-1.46 | 0.075 |
|  |  | Model 4 | 1.13 | | 0.92-1.38 | 0.253 | 1.14 | | 0.93-1.40 | 0.220 |
| Factor 3 | | Model 1 | 0.88 | | 0.68-1.15 | 0.348 | 0.88 | | 0.68-1.15 | 0.348 |
|  |  | Model 2 | 0.80 | | 0.60-1.07 | 0.137 | 0.80 | | 0.60-1.07 | 0.137 |
|  |  | Model 3 | 0.82 | | 0.62-1.09 | 0.172 | 0.82 | | 0.62-1.09 | 0.171 |
|  |  | Model 4 | 0.84 | | 0.62-1.12 | 0.229 | 0.84 | | 0.63-1.12 | 0.237 |
| Factor 4 | | Model 1 | 1.30 | | 1.10-1.54 | 0.002 | 1.30 | | 1.10-1.54 | 0.002 |
|  |  | Model 2 | 1.29 | | 1.08-1.53 | 0.004 | 1.29 | | 1.08-1.53 | 0.004 |
|  |  | Model 3 | 1.27 | | 1.07-1.51 | 0.006 | 1.29 | | 1.08-1.53 | 0.004 |
|  |  | Model 4 | 1.27 | | 1.06-1.52 | 0.010 | 1.28 | | 1.07-1.54 | 0.007 |
| Factor 5 | | Model 1 | 0.83 | | 0.67-1.02 | 0.080 | 0.83 | | 0.67-1.02 | 0.080 |
|  |  | Model 2 | 0.78 | | 0.63-0.97 | 0.027 | 0.78 | | 0.63-0.97 | 0.027 |
|  |  | Model 3 | 0.81 | | 0.65-1.01 | 0.061 | 0.79 | | 0.64-0.99 | 0.038 |
|  |  | Model 4 | 0.82 | | 0.66-1.03 | 0.090 | 0.80 | | 0.64-1.01 | 0.056 |
| Factor 6 | | Model 1 | 1.04 | | 0.90-1.20 | 0.638 | 1.04 | | 0.90-1.20 | 0.638 |
|  |  | Model 2 | 1.05 | | 0.91-1.20 | 0.502 | 1.05 | | 0.91-1.20 | 0.502 |
|  |  | Model 3 | 1.04 | | 0.91-1.20 | 0.561 | 1.05 | | 0.91-1.20 | 0.512 |
|  |  | Model 4 | 1.05 | | 0.92-1.21 | 0.470 | 1.06 | | 0.92-1.22 | 0.396 |

Model 1: Uni-variable model; Model 2: Multi-variable model, adjusted for age, gender, smoking, diabetes duration, weight, height; Model 3: Multi-variable model, further adjusted for SBP, DBP, HbA1c, HDL-C, LDL-C, and TG; Model 4: Multi-variable model, further adjusted for aspirin, antidiabetic drugs, lipid lowering drugs, antihypertensive drugs, cardiovascular disease, diabetic nephropathy, diabetic retinopathy and diabetic peripheral neuropathy. *OR* odds ratio, *CI* confidence interval.

Table S2 Association of individual PFAA with infectious event after median imputation and multiple imputation to missing value of glycated hemoglobin, triglyceride, high-density lipoprotein cholesterol and low-density lipoprotein cholesterol

|  |  | |  | Median imputation | | | Multiple imputation | | |
| --- | --- | --- | --- | --- | --- | --- | --- | --- | --- |
| Model | | Factor | PFAA | OR | 95%CI | P | OR | 95%CI | P |
| Factor 4 | | Orn | Model 1 | 1.01 | 1.00-1.01 | 0.107 | 1.01 | 1.00-1.01 | 0.107 |
|  |  |  | Model 2 | 1.01 | 1.00-1.01 | 0.127 | 1.01 | 1.00-1.01 | 0.127 |
|  |  |  | Model 3 | 1.01 | 1.00-1.01 | 0.153 | 1.01 | 1.00-1.01 | 0.123 |
|  |  |  | Model 4 | 1.01 | 1.09-1.01 | 0.188 | 1.01 | 1.09-1.01 | 0.140 |
| Factor 4 | | Asp | Model 1 | 1.01 | 1.00-1.03 | 0.078 | 1.01 | 1.00-1.03 | 0.078 |
|  |  |  | Model 2 | 1.01 | 1.00-1.03 | 0.071 | 1.01 | 1.00-1.03 | 0.071 |
|  |  |  | Model 3 | 1.01 | 1.00-1.03 | 0.059 | 1.01 | 1.00-1.03 | 0.056 |
|  |  |  | Model 4 | 1.01 | 1.00-1.03 | 0.105 | 1.01 | 1.00-1.03 | 0.101 |
| Factor 4 | | Glu | Model 1 | 1.00 | 1.00-1.01 | 0.128 | 1.00 | 1.00-1.01 | 0.128 |
|  |  |  | Model 2 | 1.00 | 1.00-1.01 | 0.197 | 1.00 | 1.00-1.01 | 0.197 |
|  |  |  | Model 3 | 1.00 | 1.00-1.01 | 0.172 | 1.00 | 1.00-1.01 | 0.138 |
|  |  |  | Model 4 | 1.00 | 1.00-1.01 | 0.234 | 1.00 | 1.00-1.01 | 0.187 |
| Factor 5 | | Hcy | Model 1 | 1.37 | 1.13-1.66 | 0.001 | 1.37 | 1.13-1.66 | 0.001 |
|  |  |  | Model 2 | 1.37 | 1.13-1.66 | 0.002 | 1.37 | 1.13-1.66 | 0.002 |
|  |  |  | Model 3 | 1.33 | 1.09-1.62 | 0.004 | 1.36 | 1.12-1.66 | 0.002 |
|  |  |  | Model 4 | 1.33 | 1.08-1.64 | 0.007 | 1.37 | 1.11-1.69 | 0.004 |
| Factor 5 | | Pip | Model 1 | 1.00 | 0.99-1.00 | 0.222 | 1.00 | 0.99-1.00 | 0.222 |
|  |  |  | Model 2 | 1.00 | 0.99-1.00 | 0.643 | 1.00 | 0.99-1.00 | 0.643 |
|  |  |  | Model 3 | 1.00 | 0.99-1.00 | 0.468 | 1.00 | 0.99-1.00 | 0.523 |
|  |  |  | Model 4 | 1.00 | 0.99-1.00 | 0.555 | 1.00 | 0.99-1.00 | 0.645 |

Model 1: Uni-variable model; Model 2: Multi-variable model, adjusted for age, gender, smoking, diabetes duration, weight, height; Model 3: Multi-variable model, further adjusted for SBP, DBP, HbA1c, HDL-C, LDL-C, and TG; Model 4: Multi-variable model, further adjusted for aspirin, antidiabetic drugs, lipid lowering drugs, antihypertensive drugs, cardiovascular disease, diabetic nephropathy, diabetic retinopathy and diabetic peripheral neuropathy. *OR* odds ratio, *CI* confidence interval, PFAA plasma free amino acid, *Orn* Ornithine, *Glu* Glutamate, *Asp* Aspartate, *Pip* Piperamide, *Hcy* Homocysteine.

**Correlation Between Individual Amino Acids in Factor 4 , Factor 5 and Inflammatory Indicators in T2DM with Infection**

Pearson's and Spearman’s correlation analysis suggested that aspartate was positively correlated with white blood cell count (Pearson's correlation coefficient = 0.09), glutamate (Pearson's correlation coefficient = 0.15) and piperamide (Pearson's correlation coefficient = 0.10). Piperamide was positively correlated with neutrophil count (Pearson's correlation coefficient = 0.08) but negatively correlated with lymphocyte count (Pearson's correlation coefficient = -0.08). (Table S3).

Table S3 Pearson's or Spearman's correlation coefficient between individual amino acids in factor 4 , factor 5 and inflammatory indicators in T2DM with infectious complications

|  | White blood cell count | | neutrophil count | | lymphocyte count | |
| --- | --- | --- | --- | --- | --- | --- |
|  | Coefficient | P value | Coefficient | P value | Coefficient | P value |
| Orn | 0.00 | 0.945 | 0.03 | 0.361 | -0.03 | 0.330 |
| Asp | 0.09 | 0.004* | 0.06 | 0.066 | -0.04 | 0.155 |
| Glu | 0.15 | <0.001* | 0.02 | 0.439 | -0.06 | 0.071 |
| Hcy | -0.04 | 0.188 | 0.01 | 0.820 | -0.03 | 0.287 |
| Pip | 0.10 | 0.002* | 0.08 | 0.009* | -0.08 | 0.007* |

Orn, Ornithine; Glu, Glutamate; Asp, Aspartate; Pip, Piperamide; Hcy, Homocysteine.

*P-values <0.05.
